# Myeloid-Derived Suppressor Cells as a Regulator of Immunity in Organ Transplantation

**DOI:** 10.3390/ijms19082357

**Published:** 2018-08-10

**Authors:** Tsukasa Nakamura, Hidetaka Ushigome

**Affiliations:** Department of Organ Transplantation and General Surgery, Kyoto Prefectural University of Medicine, Kyoto 602-8566, Japan; ushi@koto.kpu-m.ac.jp

**Keywords:** myeloid-derived suppressor cells, organ transplantation, tolerance, regulatory T cells, regulatory B cells, iNKT cells, regulatory dendritic cells, regulatory macrophages

## Abstract

Regulation of allo-immune responses is proposed as a topic for investigation in the current field of organ transplantation. As a regulator, regulatory T cells (Tregs) have received attention due to their ability to control allograft rejection. Concurrently, however, the independent action of Tregs is not enough to achieve tolerance status in many situations. Meanwhile, as a multi-functional regulator, myeloid-derived suppressor cells (MDSCs) can suppress effector T cells as well as induce Tregs or regulatory B cells (Bregs) in certain circumstances. Furthermore, the importance of a crosstalk between MDSCs and natural killer T cells to induce tolerance has been reported. Thus, orchestration between MDSCs, myeloid regulators, T/Bregs and other lymphoid/myeloid regulators can shed light on achieving allogeneic tolerance. Here, we review the current knowledge in terms of immunological regulatory function displayed by MDSCs in the context of organ transplantation. Ideal control of MDSCs would lead to a reduction of allograft rejection and subsequent long-term allograft acceptance.

## 1. Introduction

The importance of MDSCs was originally identified in the field of tumor immunity. Specific hematopoiesis was identified in tumor-bearing hosts, for example an increased proportion of monocytes with T cell-suppressing function, which were programmed as bone marrow suppressor cells [1]. In human cancer research, induced myeloid cells were certainly effective in suppressing host immunity [2]. However, the etiology of these suppressor cells was poorly understood. In tumor immunology only a decade ago, these cells were thought to be Gr-1^+^/CD11b^+^(CD115^+^) MDSCs [3,4].

During the same period, the organ transplantation field identified an important role for MDSCs in host immunity. Dugast et al. first reported the importance of MDSCs in tolerance induction in a rat kidney transplant model [5]. They identified an accumulation of CD80/CD86^+^ myeloid origin cells in an anti-CD28 Abs tolerance induction model. After this seminal study identified the significance of MDSCs in transplantation, the therapeutic potential and immune-modulatory effects of MDSCs have been widely investigated. Another study identified that Gr-1^+^/CD11b^+^/CD115^+^ MDSCs were required for tolerance induction with anti-CD40 and donor splenocytes transfer [6]. This research suggested that MDSCs were prerequisite factor to establish transplant tolerance. An additional report suggested that MDSCs transfer ameliorated allograft rejection. Although this study was not specifically aimed at understanding tolerance induction, this result suggests a therapeutic potential for MDSCs in allograft rejection, similar to mesenchymal stem cells in graft-versus host disease [7,8]. Interestingly, the interaction between MDSCs and Tregs has largely been investigated in the context of transplantation [5,6,9,10]. Collectively, these studies suggest that MDSCs have a crucial role in organ transplantation, which may be due in large part to interaction with Tregs. Studies of MDSCs in the context of transplantation are summarized in Table 1. Most rodent models of research on organ transplantation and MDSCs are mouse models, while rat models are limited. Most MDSCs research in the transplantation field has focused on MDSCs involvement in tolerance induction, mechanisms of suppression, and expansion of MDSCs. On the other hand, in human transplantation, there are only a few reports that describe MDSCs kinetics and the interrelationship between MDSCs and Tregs, especially in kidney transplantation.

In the present review, we discuss the role of MDSCs in tolerance induction with particular attention to the relationships between MDSCs and other immune cells, mechanisms of MDSCs expansion and induction, and potential clinical applications of MDSCs therapy.

## 2. Definition and Characteristics of MDSCs

MDSCs are often presented as heterogeneous cell population consisting of immature myeloid cells, which then differentiates into monocytes, dendritic cells and neutrophils. Certain pathological conditions prevent these differentiations, such as malignant tumors [30], autoimmune disease [31], or allograft rejection, resulting in MDSC expansion. MDSCs are usually divided into at least two subsets, granulocytic MDSCs (G-MDSCs) and monocytic MDSCs (M-MDSCs) [32]. In mice, MDSCs are defined as Gr-1^+^/CD11b^+^ cells with immune suppressive functions. G-MDSCs express the Ly6G surface marker, and M-MDSCs express the Ly6C surface markers. Importantly, anti Gr-1 antibodies recognize both Ly6G and Ly6C due to the high homology of these surface molecules [33]. It has been proven that these MDSCs subsets play different roles [32], but act cooperatively in tumor metastatic cascade [34]. It is still uncertain that orchestration of G- and M-MDSCs effectively prevent rejection in transplantation. In addition to these popular molecules, other cell markers such as CD49d [35], and CD115 [6], are recognized as additional markers of MDSCs. In rat, it has been reported that these MDSCs express CD11b/c and myeloid cell marker His48 [36].

On the other hand, in humans, MDSCs are characterized as CD33^+^/CD11b^+^/HLA-DR^−^ cells that suppress immune response [9,32,37,38]. There are also two distinct subsets, as with mouse MDSCs. CD14^+^/CD15^−^ are considered monocytic MDSCs. Contrastingly, G-MDSCs have a CD14^−^/CD15^+^ phenotype. In vitro functional analysis is performed to MDSCs immune suppressive activities in order to distinguish MDSCs from other myeloid cells. Furthermore, Lin^−^/CD33^+^/HLA-DR^−^ cells are categorized as early stage MDSCs (e-MDSCs), as this cell population includes immature precursors of MDSCs [39].

## 3. Effector of MDSCs

### 3.1. Inducible NO Synthase (iNOS)

iNOS is crucial for MDSCs-mediated immune suppressive functions. iNOS converts l-arginine to NO, which impairs T cell proliferation by various mechanisms, such as inducing apoptosis [40,41], suppressing T cell mitogenic responses [42], or inhibiting MHC class II expression [43]. These key roles of iNOS were identified in a number of studies regarding the role of MDSCs in transplantation. In the context of transplantation, activated MDSCs induce iNOS, provided that MDSCs contacted stimulated T cells. In a mixed lymphocytes context, an iNOS inhibitor impaired MDSCs function when T cells were stimulated by anti-CD3/CD28 Abs [5,10], or stimulator cells [7] in vitro. Immunohistochemistry was used to confirm induction of NOS under specific tolerance circumstances in vivo [5,6,10]. Interestingly, in vivo administration of the iNOS inhibitor L-NMMA impaired tolerance induction and reduced graft survival [5,10]. Although L-NMMA treatment did not specifically inhibit graft-infiltrating MDSC function, these methods implied a central role for iNOS in MDSC-mediated tolerance induction. To further assess the role of iNOS in graft-infiltrating MDSCs, it would be helpful to perform adoptive transfer with Nos2-deficient MDSCs and determine if this affected graft survival. A previous study in fact identified that MDSCs derived from *iNOS*^−/ −^ mice were not able to protect pancreatic islet cells in an MDSC and islet co-transplantation model [7]. These studies support iNOS contribution in MDSCs-mediated function.

### 3.2. Arginase

Arginase is an enzyme that hydrolyzes arginine into ornithine and urea. Arginase also inhibits T cell proliferation, and is known to play a significant role in the immune response [44]. Because arginase and iNOS have arginine as a common substrate, the function of these two enzymes is similar; both enzymes deplete the essential amino acid l-arginine, resulting in immunomodulation. In the context of cancer research, arginase-overexpressing MDSCs inhibited T cell cytotoxic function. Further, blocking induction of arginase by COX-2 inhibitors increased the anti-tumor effect of lymphocytes [45]. In contrast, functional MDSCs had high arginase activity after effective immunosuppression [10] or tolerance induction [6] prolonging the allograft survival. Thus, MDSCs with increased expression of arginase might contribute to protection of allografts in organ transplantation. Overall, arginase is a critical component of MDSCs.

### 3.3. Reactive Oxygen Species (ROS)

ROS also play a role in the immune suppressive function of MDSCs. A human kidney transplant study suggested that intracellular ROS function was a crucial factor of MDSCs as a regulator of acute rejection. S100A8 and S100A9, which enhance ROS production, act as stimulators of inflammation, but also as modulators of adoptive immunity. In peripheral blood mononuclear cell (PBMCs) and intra-graft cells, S100A8 and S100A9 expression were positively correlated with MDSCs CD33^+^ mRNA levels. High expression of S100A8 and S100A9 was correlated to better graft outcomes [28]. It is also true, therefore, ROS is one of the important effectors of MDSCs, although reports of MDSCs with ROS involvement in organ transplantation are still limited.

### 3.4. Indoleamine-2,3-Dioxygenase (IDO)

IDO is a heme enzyme that catabolizes tryptophan to kynurenine, which induces T cell anergy [46]. IDO is usually produced by dendritic cells (DCs), and induces DCs-derived Tregs. IDO expression in MDSCs also plays an important role in suppressing T cell proliferation [47]. A study in a cell-based xenogenic cytotoxicity model identified that MDSCs suppress macrophage-mediated cytotoxicity through IDO expression. [48]. Another study, described in detail below, demonstrated that IDO expression is enhanced by CsA, and plays a key role in MDSCs-mediated immunosuppression [49]. In a cardiac transplantation model, infusion of donor splenocytes treated with the chemical cross-linker 1-ethyl-3-(3′-dimethylaminopropyl)-carbodiimide (ECDI) (ECDI-SP) expanded Ly6C^high^ MDSCs. This study identified that MDSCs-mediated immune suppression is dependent on IFN-γ, and its downstream effectors IDO and iNOS [17]. On the other hand, interestingly, IDO also controls MDSCs expansion. In a tumor model, Holmgaard et al. [50] reported that IDO inhibition reduces the number of MDSCs in tumors. Taken together, IDO is an important effector as well as inducer of MDSCs.

### 3.5. PD-L1

Due to recent advancements in cancer research, PD-1 and PD-L1 interactions have been a topic of intense investigation because these molecules are possible therapeutic targets [51]. It is well known that PD-L1 expression on tumor cells and supportive cells in the tumor microenvironment strongly suppresses of T cell immunity [52]. In the tumor microenvironment, MDSCs express high baseline PD-L1, which impedes the effect of irradiation on tumor tissue [53]. As a supportive component of the tumor microenvironment, MDSCs express PD-L1 to suppress T cell immunity [54]. In the context of organ transplantation, the PD-L1/PD-1 axis is important in prolonging graft survival. PD-L1Ig and anti-CD40L treatment achieved long-term graft survival (median graft survival > 140 days) in an islet transplantation model [55]. Further, PD-L1 expression on MDSCs also has been confirmed, and results in immunosuppression in a cardiac transplantation model [21]. Taken together, it can be argued that the PD-L1/PD-1 axis plays a key role in MDSCs-mediated transplant tolerance induction.

### 3.6. IL-10

IL-10 suppresses the CD28 signaling pathway by inhibiting tyrosine phosphorylation of CD28, and thus is considered an immune-modulatory cytokine [56]. In fact, there are many studies demonstrating that IL-10 plays an important role in tolerance induction, as expertly reviewed previously [57,58]. Generally, IL-10 is produced by monocytes, helper T cells, B cells or mast cells. MDSCs are also capable of producing IL-10, resulting in T cell suppression and Treg induction [59]. IL-10 production may therefore contribute to the immune suppressive function of MDSCs.

## 4. Histologic Localization to Elucidate MDSCs Function

It is well known that cell-to-cell contact is essential in MDSC suppression of host immunity [60]. Especially in organ transplantation, the migratory capacity of MDSCs is prerequisite in their regulatory role. Garcia et al. [6] demonstrated the importance of MDSCs migration into transplantation allograft utilizing MDSCs from mice deficient in various components of cellular migration. CCR2-deficient, P/E selectin-deficient, and fucosyltransferase IV–VII double-deficient MDSCs transfer failed to establish transplant tolerance, which suggests that the immune regulatory function of MDSCs occurs through direct contact with other cells rather than through paracrine mechanisms. This may explain in part why intravenous administration of MDSCs was ineffective in achieving allograft survival [5]. To transfer MDSCs to the site of immune regulation, delivery through inflow vessels may be a more viable approach [10]. Following transplantation with immunosuppression, the expressions of chemokine receptors on MDSCs were up-regulated both in murine models [18] and in humans [29]. Thus, chemokine receptors related with cellular migration are of vital importance for true recruitment of MDSCs.

## 5. Inducers of MDSCs

**Figure 1:**
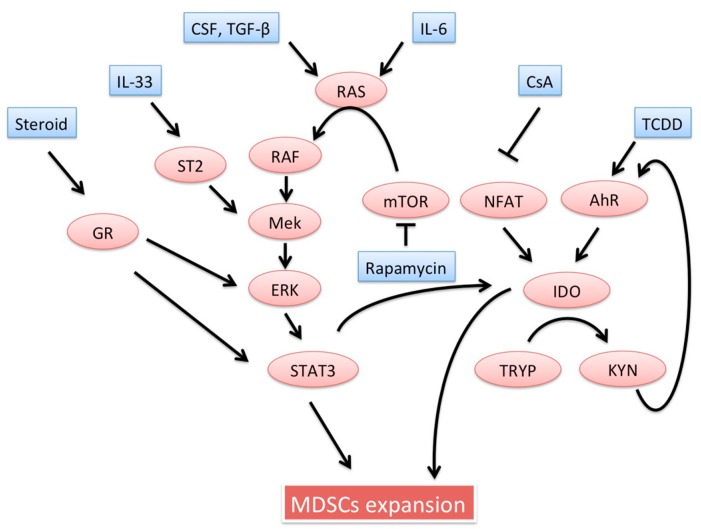
MDSCs expansion is regulated by several factors in vivo microenvironment. This figure summarizes possible MDSCs inducers described in the context of transplantation and their signaling pathways. RAS is a target GTPase of CSF, TGF-β, or IL-6. mTOR inhibitor, rapamycin, paradoxically activates the RAS/RAF/MEK/ERK pathway. This pathway locates at a downstream of IL-33 and Steroid signaling pathways. Both cyclosporin and TCDD induce IDO through NFAT and aryl hydrocarbon receptor, respectively. The STAT 3 is mainly involved in MDSCs expansion as well as IDO expression. MDSCs, myeloid-derived suppressor cells; CSF, colony stimulating factor; IDO, indoleamine-2,3-dioxygenase; TCDD, 2,3,7,8-tetrachlorodibenzo-*p*-dioxin; NFAT, nuclear factor of activated T cells; STAT, signal transducer and activator of transcription.

MDSCs induction is required to prevent host allograft rejection. Several studies have shown that tolerance induction regimens or certain drugs are dependent on induction of functional MDSCs. Importantly, in the literature, “induction” of MDSCs refers to one or more specific interactions. Generally, induction of MDSCs indicates: (i) an increase in the absolute number of MDSCs; (ii) reinforcement of MDSCs effectors; (iii) up-regulation of molecules regulating MDSCs migration; or (iv) enhanced ability of MDSCs to induce other regulatory cells. Below, potential inducers of MDSCs are introduced. Detailed signaling pathways possibly involved in MDSCs expansion were already well documented [32]. Interestingly, many possible inducers seem to stimulate the Ras/Raf/MEK/ERK pathways, resulting in the signal transducers and activators of transcription (STAT) 3 activation. These inducers involved in MDSCs recruitment are schematically described in Figure 1.

### 5.1. Induction Regimens

In vivo expansion of MDSCs is clinically important in human transplantation, and examining requirements for MDSCs induction could lead to approaches for effective in vivo MDSCs expansion. Moreover, potential means to prepare activated MDSCs ex vivo to rescue allografts from rejection have significant therapeutic potential. In preclinical settings, there are several regimens used to induce MDSCs from bone marrow [7,61] or induced pluripotent stem cells [62] in vitro. First, colony stimulating factor (CSF) seems to play important role in induction of bone marrow-derived MDSCs. Therefore, GM-CFS (8–20 ng/mL) is routinely used in these regimens. In addition to CSF, TGF-β also seems to be important to generate MDSCs with iNOS or arginase [63]. Notably, both CSF and TGF-β have an ability to activate the Ras/Raf/MEK/ERK pathway [64,65]. In some cases, M-CFS, IL-4, and immune stimulators such as lipopolysaccharide or IFN-γ are used [7]. Although there are slight differences depending on the protocol, MDSCs can be efficiently generated in vitro in 3–14 days.

### 5.2. Medications

#### 5.2.1. Steroids

Several basic research studies demonstrated that dexamethasone induces MDSCs in transplantation models [18,23,24]. Furthermore, steroid treatment induces leukocytosis in transplant recipients in clinical settings. Clinical studies have suggested that MDSCs increase in PBMC after surgery [9,29]. Almost all transplant recipients received steroids in addition to other immunosuppressants. Okano et al. [29] identified that exogenous steroid dose positively correlated with the absolute number of MDSCs in transplant recipients. In an experimental model of organ transplantation, dexamethasone administration recruited iNOS expressed MDSCs, while blocking glucocorticoid receptors by using RU-486 reduced CXCR2 expression and recruitment of MDSCs [18]. This suggested that the glucocorticoid-glucocorticoid receptor axis may play a role in MDSCs recruitment. Overall, there seems to be a consensus that steroid administration has the potential to expand MDSCs, and induce MDSCs iNOS expression, resulting in NO production and T cell suppression, though detailed mechanisms by which steroids potentiate MDSCs still remain unclear.

#### 5.2.2. mTOR Inhibitor: Rapamycin

Initially, mTOR was documented in 1991 as a target of rapamycin in *Saccharomyces cerevisiae*. Clinically, mTOR inhibitors are the main immunosuppressive drugs administered to organ transplant recipients [66]. However, the mechanisms by which mTOR inhibitors suppress immunity are incompletely understood. Interestingly, administration of the mTOR suppressor rapamycin plays a crucial role in inducing MDSCs [10,67,68]. Since there is a mTORC1-MAPK feedback loop [69], it can be considered that simple mTOR inhibition induces activation of the MEK/ERK signaling pathway, which causes blunt immune responses where MDSCs are recruited and functionally suppress rejection. However, an opposing report suggested that mTOR inhibition by rapamycin reduced M-MDSCs infiltration into the allo-skin grafts. Furthermore, to prove direct action of mTOR on M-MDSCs, mice with myeloid-specific deletion of mTOR were used, demonstrating that deletion of mTOR in myeloid cells decreases M-MDSCs in skin transplantation [70]. Differential transplantation models or rapamycin dosages may explain in part these conflicting results. Although the results were not congruent, these studies clearly demonstrated that mTOR affects MDSCs recruitment, although the effect may be context-dependent.

#### 5.2.3. Calcineurin Inhibitor: Cyclosporine

The calcineurin inhibitor, cyclosporine A or Tacrolimus, is at the core of currently available immunosuppressive drug regimens. In a cyclosporine-treated organ transplantation model, functional MDSCs are an important regulator for prevention of allograft rejection [49]. In this model, expression of NFATc1 in MDSCs was diminished, shifting the Th2-Th1 balance towards Th2, and decreasing IFN-γ production in CD8^+^ T cells. Inhibition of NFAT up-regulated IDO in MDSCs, subsequently enforcing the immunosuppressive function of MDSCs. Thus, the importance of cyclosporine-NFAT-IDO axis in MDSCs was suggested in the setting of skin transplantation.

#### 5.2.4. IL-6

Originally, IL-6 was discovered as a cytokine that regulates differentiation of B cells into plasma cells [71]. Since this seminal discovery, many other immune regulatory effects of IL-6 have been identified, including regulation of MDSCs. In mammary tumor models, IL-1β and IL-6 promote accumulation of MDSCs and contribute to tumor progression [72]. In ex vivo cell culture, IL-6 is a potent inducer of MDSCs in reaction to TGFβ [63]. In the human transplantation field, IL-6 is positively correlated to MDSCs expansion [29]. Together, this evidence suggests that IL-6 positively regulates MDSCs, although the specific mechanism has not yet been identified.

#### 5.2.5. IL-33

IL-33 is also referred to as nuclear factor from high endothelial venules, and belongs to the IL-1 family. IL-33 is a ligand for ST2, activating NF-κB and MAPK signaling pathways [73]. The IL-33/ST2L axis promoted generation of MDSCs, prolonging allograft survival in a mouse heart transplant model [12]. Interestingly, depletion of MDSCs by anti-Gr-1 (RB6-8C5) did not affect the graft survival, while the administration of anti-CD25 antibodies (Tregs depletion) shortened survival. Although this research did not show MDSCs contribution for graft survival prolongation, these results demonstrated IL-33 had an ability to modulate myeloid population, differentiating toward MDSCs.

#### 5.2.6. 2,3,7,8-Tetrachlorodibenzo-*p*-dioxin (Aryl Hydrocarbon Receptor Agonist)

Exposure to 2,3,7,8-Tetrachlorodibenzo-*p*-dioxin (TCDD) may lead to expansion of MDSCs [74]. The aryl hydrocarbon receptor (AhR) has recently gained popularity, as down-stream signaling pathways regulate myeloid and T cell differentiations [75,76]. These possible immunomodulation abilities in transplantation were recently reviewed elsewhere [77] although the relationship between AhR and MDSCs was not discussed. Interestingly, there is a close relationship between AhR and IDO, as kynurenine is an agonist of AhR [78]. In the tumor microenvironment, IDO expression was observed in MDSCs, and a significantly high kynurenine/tryptophan ratio was confirmed [79]. Although there is still no concrete evidence that AhR is positively linked to MDSCs expansion, the potential relationship between AhR and MDSCs might shed light on currently unexplained involvement of environmental factors.

#### 5.2.7. Hepatic Stellate Cells

Hepatic stellate cells (HSCs) are a liver mesenchymal cells generally involved with liver regeneration and fibrosis [80]. Interestingly, it has been reported that HSCs accelerate MDSCs differentiation even from monocytes [81]. By applying these findings, it was confirmed that HSCs-induced MDSCs were effective to alleviate rejection and prolong islet graft survival [7]. In liver transplantation, donor-derived HSCs are inevitably transferred to recipients and contact with recipient-derived monocytes in the allograft [82]. Thus, this situation might simulate HSCs-induced MDSCs generation in vivo. It is worth investigating the relationship between HSCs and MDSCs in terms of tolerance induction.

## 6. Inhibitors of MDSCs

Negative feedback systems in MDSCs are important to prevent over activation of immunosuppression in vivo. Currently, research regarding inhibition of MDSCs is mainly limited to the cancer field, as this could have significant therapeutic potential. Agents that seem to be unfamiliar to transplantation fields are excluded in this section. Those agents were already well reviewed [32]. However, over activation of immunosuppression may also be relevant to the transplant field, as this could result in complications such as severe infection or tumor development. Thus, to control MDSCs in vivo, MDSCs inhibitors should be studied in the context of the organ transplantation field. MDSCs inhibitors can be categorized as differentiation inducers, anti-cell surface marker antibodies, or effector inhibitors.

### 6.1. All-Trans Retinoic Acid (ATRA)

ATRA was originally discovered as a potent agent able to induce complete remission of acute myeloid leukemia (M3). ATRA has a potential to induce MDSCs differentiation into mature myeloid cells. Although there are several reports that ATRA can antagonize MDSCs in the field of cancer research [83,84,85], the potential utilization of ATRA in the context of organ transplantation has not yet been investigated. Further research in this area would be expected.

### 6.2. MEK Inhibitor

The MEK inhibitor trametinib was originally developed as an anticancer drug that prevents MEK phosphorylation and downstream proliferative signaling pathways [86,87]. Because colony stimulating and/or vascular endothelia growth factors can induce MDSCs, activation of the Raf/MEK/ERK pathway is likely important in controlling MDSCs kinetics. Thus, it is reasonable to believe that stabilization of MEK may prevent MDSCs activation. This theory was proven in both transplantation [10], and cancer research [88]. In fact, a MEK inhibitor clearly abrogated MDSCs expansion. Thus, MEK inhibition may be important mechanistically and therapeutically in negatively regulating MDSCs expansion.

### 6.3. Anti-PD-L1 Antibodies

PD-L1 is considered an important ligand for MDSCs in suppression of immunity, as mentioned above. Thus, anti-PD-L1 antibodies deplete some populations of MDSCs [53]. As the number of transplant patients as well as the availability of immune checkpoint antibodies for malignant tumors increases [89], the incidence of recipients who also received anti-PD-L1 treatment for cancer is expected to increase. Transplant tolerance would be compromised by anti-PD-L1 therapy, which may be due in part to MDSCs depletion [90]. Anti-PD-L1 treatment and other cancer immunotherapies will therefore be an important clinical consideration in transplant recipients in the future.

### 6.4. IDO Inhibitors

IDO is considered as an important effector of MDSCs, as discussed above. In a PD-L1 resistant lung cancer model, high IDO expression was observed in MDSCs. Inhibition of IDO restored T cell anti-tumor activity, while the percentage of IDO^+^ MDSCs was reduced [91]. These results also confirm the participation of IDO in MDSCs induction, which would be applicable in organ transplantation.

## 7. Isolation Techniques

Isolation of MDSCs is required for the cell therapy, excluding in vivo expansion where functional MDSCs exist in transplant recipients. In mice, G-MDSCs (Ly6G^+^/Ly6C^low^/CD11b^+^) and M-MDSCs (Ly6G^−^/Ly6C^high^/CD11b^+^) can be isolated from culture, splenocytes, or bone marrow by using biotinylated antibodies against Ly6G and Gr-1, magnetic microbeads, and magnetic-activated cell sorting columns (MACS) (Miltenyi Biotec, Auburn, CA, USA). Alternatively, a flow cytometric cell sorter (FACS) is also available for capturing these cell surface markers. In the same manner, in humans, CD33^+^/CD11b^+^/HLA-DR^−^ MDSCs are also isolated using MACS or FACS. Either way, it is prudent to enrich MDSCs to a certain degree prior to the cell sorting by using inducers.

## 8. Relationships between MDSCs and Other Immune Cells

**Figure 2:**
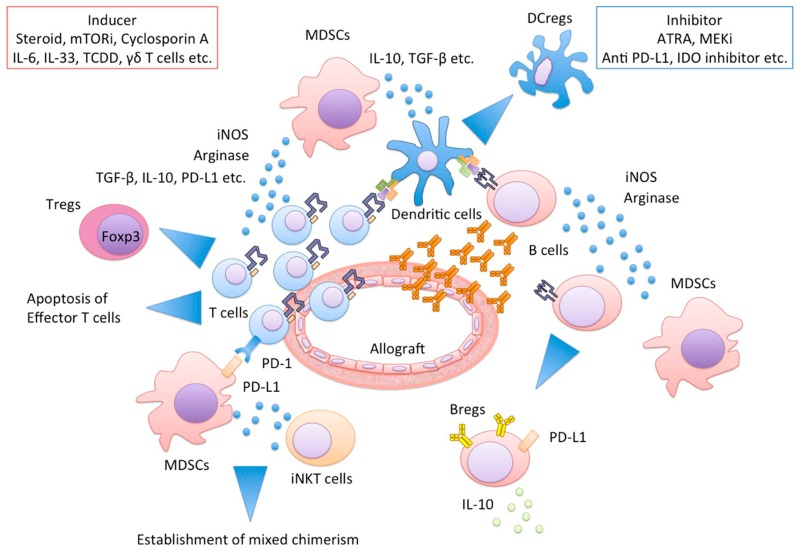
MDSCs and their networks cooperatively protect allograft injury. Functional MDSCs produce a large amount of iNOS or arginase, resulting in effector T cell apoptosis, Tregs and Bregs and DCregs induction. MDSCs and invariant NKT cells interactions regulate the establishment of mixed chimerism. MDSCs, myeloid-derived suppressor cells; iNOS, inducible NO synthase; Tregs, regulatory T cells; Bregs, regulatory B cells; DCregs, regulatory dendritic cells; NKT, Natural Killer T cells.

It has been recognized that MDSCs have a wide variety of networks with other immune cells, as described in Figure 2. As a result, host immune system towards immune suppressive and tolerogenic status. Herein, we present relationships between MDSCs and representative immune cells: T cell, DCs, Macrophages, B cell, NKT cells, and γδ T cells.

### 8.1. MDSCs and Tregs

The importance of Tregs in immune regulation is widely recognized in cancer, autoimmune disease, inflammation, and allograft rejection [92]. Among these conditions, it has been reported that MDSCs maintained development of Tregs in tumor-bearing mice, and that this immune reaction suppressed host anti-tumor responses [4]. The interrelationship of MDSCs and Tregs is an intense topic of investigation, as these cells have an orchestrated ability to suppress host immunity. This relationship has been studied not only in cancer research, but also in the fields of organ transplantation and autoimmunity. A large proportion of MDSC research in the context of organ transplantation is focused on this relationship. MDSCs are likely to have differential effect on Tregs and effector T cells were reported. MDSCs completely inhibit the effector T cell proliferation, but only partially suppress Tregs in vitro [5]. This effect was also observed in vivo in a cardiac transplantation model. This research highlighted the notion that MDSCs are required for Treg development using tolerance inducing Foxp3-RFP mice [6]. Similar results were also confirmed by functional MDSCs transfer transplantation model [21]. In terms of mechanisms, it has been reported that TGF-β played a central role in MDSCs mediated Tregs induction [93]. In addition to Tregs induction from CD4^+^/CD25^−^ T cells, MDSCs derived TGF-β also offered Tregs plasticity that Th17 cells transdifferentiated into Foxp3^+^ Tregs [94]. Furthermore, IL-10 and PD-L1 are also considered as important elements to expand Tregs [59,95]. Although the detailed mechanism by which MDSCs induce Tregs is still unclear, in human transplantation, a positive correlation between MDSCs and Tregs was observed [9,29]. Inducing MDSCs generally takes relatively shorter periods of time both in vitro and in vivo, while mature Tregs development usually needs longer duration. Thus, MDSCs might play a crucial role in the early phase following transplantation, followed by Tregs immune regulation. In fact, antigen-specific Tregs do not have a significant regulatory function in the early stages of transplantation, as the adaptive immune response is not active in acute contexts [96]. From this point of view, fully matured Treg transfer is attractive in conjunction with MDSCs transfer, in addition to Treg induction in vivo. In fact, in a pilot study of human liver transplantation [97], ex vivo-generated Tregs derived from donor lymphocytes were transferred to recipients on Post-Operative Day 13. This study concluded that ex vivo-generated Tregs transfer is useful to minimize immunosuppressive agents, although this study did not evaluate the potential role of MDSCs. In total, it can be argued that MDSCs and Tregs orchestration potentially brings circumstances where allografts tend to be accepted with tolerance.

### 8.2. MDSCs, Dendritic Cells, and Macrophages

Because MDSCs, DCs and Macrophages are derived from the same progenitor cells, the interrelationships between these cell populations are likely to be of physiological significance. Similar to MDSCs, regulatory DCs (DCregs) and regulatory macrophages (Mregs) have the potential to ameliorate allograft rejection [98,99,100]. It is possible to induce these three regulatory myeloid cells using specific conditions, although the definitions are slightly different, depending on reports [101,102,103,104]. Given the results of these reports, IL-10^+^ or TGF-β^+^ MDSCs may positively recruit DCregs and Mregs. Although these three distinct regulatory myeloid cells are capable of immunosuppression, the mechanisms of immunosuppression seem to be cell type specific. DCregs are capable of directly inducing T cell but not Treg hyporesponsiveness, while Mregs and MDSCs recruit Tregs. Interestingly, rapamycin also potentiates the activity of DCs, activating DC induction of Tregs [105,106], similar to MDSCs [10]. Among these populations, MDSCs are most readily cultured, typically 3–4 days [61,101], compared to 7–21 days for Mregs or DCregs, so MDSCs may be most applicable as a therapeutic approach. Of these myeloid populations, MDSCs have the central regulatory role in allograft rejection, and likely function as a bridge to DCregs and Mregs.

### 8.3. MDSCs and Bregs

Immunomodulatory function of MDSCs on B cells has been recently reviewed [107]. The immunosuppressive function of B cells was first noted in the context of delayed-hypersensitivity in 1974 [108], and has recently gained popularity. Conversely, the detailed mechanisms by which B cells suppress the immune response remain incompletely understood. Park et al. [61] first reported that MDSCs induce and expand Bregs via iNOS in a murine model of SLE. This research showed that functional IL-10-producing Bregs were induced when splenocytes and MDSCs were co-cultured in vitro. This finding was also observed in vivo in the SLE model mouse. MDSC transfer increased splenic Bregs, and subsequently ameliorated SLE lesions, such as lymphocytes infiltration into the kidneys and liver. The iNOS inhibitor L-NMMA partially inhibited the Breg-inducing effects of MDSCs, demonstrating the importance of iNOS in MDSC Breg induction. More recently, it has been reported that MDSCs are capable of transforming B cells to PD-1^−^/PD-L1^+^/CD19^+^ Bregs in a breast cancer model [109]. This Breg-inducing function of MDSCs is also important in the field of organ transplantation, especially regarding immunological tolerance in antibody-mediated allograft rejection.

### 8.4. MDSCs and NKT Cells

NKT cells are of lymphoid origin, following T, B, and NK cells. NKT also have heterogenous subpopulations with differential functions. Invariant NKT (iNKT) cells can activate both innate immunity and acquired immunity, resulting in inducing long-term immunity memory [110]. Conventionally, iNKT have bene thought to suppress MDSCs, inhibiting tumor progression [111]. However, it has been demonstrated that tolerance induction by mixed chimerism required the interaction between MDSCs and IL-4 secreting iNKT cells in a combined bone marrow and heart transplant model [16]. MDSCs are therefore an essential component of mixed chimerism. In fact, mixed chimerism is a clinically available method for induction of transplant tolerance [112]. These approaches have been extensively focused in clinic as a promising tolerance induction regimen [113,114,115]. It is worth investigating MDSCs and iNKT cells interaction.

### 8.5. MDSCs and γδ T Cells

γδ T cells are a subtype of T cells that express different T cell receptors, consisting of γ and δ chains, compared with conventional αβ T cells. γδ T cells play both regulatory and proinflammatory roles, depending on the context. γδ T cells induce mobilization of MDSCs into the liver in a hepatitis B virus infection model, resulting in CD8^+^ T cell exhaustion [116]. There has been no report of the role of MDSC and γδ T cell interactions in organ transplantation. However, because γδ T cells regulate hepatic migration of MDSCs, further research would be expected in the organ transplant field.

## 9. Clinical Organ Transplantation

In clinical settings, an extensive immunosuppressive treatment is initiated from induction to around one month following transplantation, with systemic administration of several immunosuppressants even in stable cases [117,118]. Moreover, in acute rejection cases, high doses of steroid or anti-lymphocyte antibodies are usually required. Severe acute rejection cases sometimes result in poor transplant outcomes. MDSCs may be a potential therapeutic modality for acute rejection cases that are refractory to conventional immunosuppressive therapies. Furthermore, MDSCs might have a promising role in clinically available tolerance induction.

## 10. Feature Perspective and Possible Clinical Application

### 10.1. Cell. Therapy

The ideal approach to utilizing MDSCs clinically would be tolerance induction. However, as with mesenchymal stem cells for graft-versus-host disease, MDSCs transfer may be capable of ameliorating acute rejection, as has been suggested by basic research studies [7,10]. Although many cases of acute rejection are successfully managed by currently available treatments, refractory cases of acute rejection could require re-transplantation or be life-threating in some cases. For those cases, MDSC therapy may be life-saving. Potential complications such as over immunosuppression, infection, and onset of malignant tumors must still be overcome to realize this cell therapy. To address these potential complications, it will be essential to control MDSCs function adequately in humans. To this end, further research into the mechanisms of MDSCs activation and suppression is necessary.

### 10.2. Tolerance Induction “Integration MDSCs and Other Immune Cells in Organ Transplantation”

The most ideal clinical application for MDSCs therapy is tolerance induction. The complex networks between MDSCs and other immune cells are discussed above. In fact, in clinical settings, all initial ischemia–reperfusion injury (innate immunity), subsequent cellular rejection and antibody-mediated rejection (acquired immunity) should be addressed to overcome immunological graft destruction [119]. Since MDSCs can communicate with a variety of immune cells, MDSCs might have a potential to manage all aspects of immunological graft destruction. When considering these relationships in the context of in vivo transplantation, both the induction and survival times of the cell types in question should be considered. For example, MDSCs are quickly induced within few days but are short-lived, while regulatory lymphoid cells have a much longer maturation time. In fact, although MDSCs essential in establishment of immunological tolerance [6,16], total numbers of MDSCs gradually decrease and remain within normal ranges in the remote period following organ transplantation [16]. To safely apply MDSCs cell-based therapy in human transplantation, it is important to determine the timing and duration of contact. If the timing is not correct, MDSCs therapy might trigger infection or development of tumors, but have minimal beneficial effects in transplant rejection. As a method, it is reasonable to transfer MDSCs derived ex vivo at an ideal timing to avoid these consequences. Given the strategy of MDSCs-induced tolerance, it is of vital importance to establish the bridge between MDSCs in innate immunity and Tregs, Bregs, or iNKT cells in adoptive immunity.

### 10.3. Marker of Immunosuppression

Monitoring the status of immunosuppression is crucial to prevent rejection and overimmunosuppression in transplant recipients. The most common means to monitor immunosuppression status is to measure the concentration of immunosuppressive drugs including calcineurin inhibitors, everolimus, and anti-metabolic drugs [120]. Although steroid hormones are central to immunosuppressive regimens, monitoring steroid concentration does not seem to be useful. In particular, acute rejection usually requires a high dose of steroid pulse therapy. Nevertheless, a fixed dosage of steroids is routinely administered. However, clinical efficacy does not always correlate with steroid dosages or serum concentrations. Because steroids expand MDSCs and act as immunosuppressive agents, monitoring MDSCs could be used as a means to assess steroid efficacy, and steroid pulse can be tailored based on the degree of MDSC expansion. Further, there is no clinically available method to reverse the effects of steroids in the event of overimmunosuppression. MDSC inhibitors would be promising means for reversing the effects of steroids, provided that this relationship is concretely established.

### 10.4. Source for MDSCs

MDSCs have been cultured and isolated from bone marrow on a small scale. However, bone marrow aspiration is invasive, so this method of MDSCs isolation may not be clinically desirable. Alternatively, prior studies have demonstrated that leukapheresis and GM-CSF administration is an effective means to isolate MDSCs and maintain functionality in cryopreserved cells in healthy rhesus macaques [25]. In patients receiving hemodialysis or relying on an auxiliary artificial heart waiting for transplantation, this strategy could be used to cultivate the required number of MDSCs. The remaining barriers to this approach are the stability of ex vivo MDSCs and possible development of malignant tumors.

## 11. Concluding Remarks

MDSCs regulate host immunity in organ transplant recipients, suppressing host immunity to prevent allograft rejection, and this regulatory function makes MDSCs an attractive therapeutic modality. MDSCs involvement in tolerance induction in pre-clinical models has been confirmed in many reports. However, only limited data are currently available in clinical studies, which have only demonstrated the natural course of MDSC behavior and the possible relationship with Tregs. There are still unknown mechanisms by which MDSCs exhibit suppressive functions and contribute to development of tolerance status. The development of effective MDSCs induction and control is a current barrier to applying MDSCs for transplant tolerance in clinical practice. Adjusting current knowledge regarding MDSCs would lead to improved outcomes in organ transplantation.

## Figures and Tables

**Table 1:** MDSCs in transplantation.

Author	Refs.	Year	Species	Organ/Tissue	Phenotype	Possible Mechanism of Suppression	CD4+ Tregs Involvement	Inducer	Remarkable Findings
**Mouse/Rat**									
Dugast	[5]	2008	Rat	Kidney	CD6^−^/NKRP^−^1^+^/CD80^+^/CD86^+^	iNOS	+	anti CD28 Abs	Anti-CD28 Abs tolerance induction may dependent on iNOS+MDSCs. MDSC acted in a contact-dependent manner
Zhang	[11]	2008	Mouse	Skin	Gr-1^+^/CD11b^+^	Arginase	N/A	ILT2 inhibitory receptor	Adoptive transfer of generated MDSCs prolonged skin allograft survival
Garcia	[6]	2010	Mouse	Heart	Gr-1^+^/CD11b^+^	iNOS, Arginase	+	anti-CD40 Abs/DST	MDSCs migrated into the allograft prevent rejeciton and develop Tregs. Gr-1^−^/CD11b^+^ monocytes express PD-L1
Turnquist	[12]	2011	Mouse	Heart	Gr-1int/CD11b^+^	N/A	+	IL-33	IL-33 induced MDSCs, but MDSCs did not prolong allograft survival in this model
Adeegbe	[13]	2011	Mouse	Skin	Gr-1^+^/CD11b^+^	N/A	+	G-CSF, IL-2	MDSCs and Tregs down-modulatd alloreactive T-cell responses in a synergistic manner
Chen	[14]	2012	Mouse	Heart	Gr-1^+^/CD11b^+^	IDO	+	ECDI-SP	Allograft protection by ECDI-SP depended on MDSCs
Dilek	[15]	2012	Rat	Kidney	CD6^−^/NKRP-1^+^/CD80^+^/CD86^+^	N/A	+	anti CD28 Abs	MDSCs contributed to the establishment of a graft to periphery CCL5 gradient
Arakawa	[7]	2014	Mouse	Islet	Gr-1^+^/CD11b^+^	iNOS	N/A	GM-CSF, IL-4, hepatic stellate cells	In vitro generated MDSCs had an ability to protect allogeneic islet cells
Hongo	[16]	2014	Mouse	Heart	Gr-1^+^/CD11b^+^	PDL1, arginase-1	-	iNKT cells	mixed chimerism establishment required MDSCs
Bryant	[17]	2014	Mouse	Heart	Gr-1^+^/CD11b^+^	IDO, iNOS	+	ECDI-SP	MDSCs protected allografts through their own production of IFN-γ
Liao	[18]	2014	Mouse	Skin	Gr-1^+^/CD11b^+^	iNOS	N/A	dexamethasone	Glucocorticoid-glucocorticoid receptor-NO cascade was crucial by dexamethasone mediated immune suppression
Nakamura	[10]	2015	Mouse	Heart	Gr-1int/CD11b^+^	iNOS	+	rapamycin	mTOR and Raf/MEK/ERK signaling pathways play an important role in MDSC expansion
Gajardo	[19]	2015	Mouse	Skin	Gr-1low/CD11b^+^	iNOS, Arginase	+	IL-33	IL-33 target cell population during transplant rejection corresponded to MDSCs
Sido	[20]	2015	Mouse	Skin	Gr-1^+^/CD11b^+^	N/A	N/A	Delta(9)-Tetrahydrocannabinol	Delta(9)-Tetrahydrocannabinol induced MDSCs mainly through CB1 receptor
Nakamura	[21]	2016	Mouse	Heart	Gr-1^+^/CD11b^+^	iNOS	+	rapamycin	MDSCs induced Tregs expansion in allografts
Yang	[22]	2016	Mouse	Skin	Gr-1^+^/CD11b^+^	iNOS	N/A	M-CSF, TNFα	PD-L1 was upregulated on MDSCs
Zhao	[23]	2018	Mouse	Heart	Gr-1int/CD11b^+^	iNOS	+	dexamethasone	GR signaling recruited transferred MDSCs into the allograft
Nakao	[24]	2018	Mouse	Heart	Gr-1^+^/CD11b^+^	iNOS	+	dexamethasone	MDSCs regulated the expansion of Tregs
**Other**									
**Zahorchak**	[25]	2015	Macaque	N/A	CD33^+^/CD11b^+^/HLA-DR^−^	Arginase	+	GM-CSF, IL-4	availability of cryopreserved MDSCs
**Human**									
Luan	[9]	2013	Human	Kidney	CD33^+^/CD11b^+^/HLA-DR^−^	N/A	+	N/A	There was a positive correlation between the number of MDSCs and Tregs
Meng	[26]	2014	Human	Kidney	CD33^+^/CD11b^+^/HLA-DR^−^	N/A	+	N/A	MDSCs associated with higher frequency of Tregs and better graft survival
Hock	[27]	2015	Human	Kidney	CD33^+^/CD45^+^/HLA-DR^−^/(CD14^+^/CD66b^+^)	N/A	N/A	N/A	The number of MDSCs increased following the initiation of immunosuppression.
Rekers	[28]	2016	Human	Kidney	CD33^+^/CD11b^+^/(CD14^+^)	ROS	+	S100A8, 9	S100A9 expression predicted better graft outcomes
Okano	[29]	2018	Human	Intestine	CD33^+^/CD11b^+^/HLA-DR^−^/low	N/A	+	IL-6, exogenous steroid hormone	MDSCs in PBMC during rejection decreased

MDSCs, myeloid-derived suppressor cells; iNOS, inducible NO synthase; IL-T2, inhibitory receptor immunoglobulin-like transcript-2; DST, donor splenocytes transfusion; CSF, colony stimulating factor; IDO, indoleamine-2,3-dioxygenase; ECDI-SP, 1-ethyl-3-(3′-dimethylaminopropyl)-carbodiimide; GR, glucocorticoid receptor; ROS, reactive oxygen species; PBMC, peripheral blood mononuclear cells.

## Abbreviations

MDSCs	myeloid-derived suppressor cells
G-MDSCs	granulocytic MDSCs
M-MDSCs	monocytic MDSCs
e-MDSCs	early stage MDSCs
iNOS	inducible NO synthase
ROS	reactive Oxygen Species
PBMC	peripheral blood mononuclear cell
IDO	indoleamine-2,3-dioxygenase
DCs	dendritic cells
ECDI	1-ethyl-3-(3′-dimethylaminopropyl)-carbodiimide
ECDI-SP	donor splenocytes treated with 1-ethyl-3-(3′-dimethylaminopropyl)-carbodiimide
STAT	signal transducers and activators of transcription
CSF	colony stimulating factor
TCDD	2,3,7,8-Tetrachlorodibenzo-*p*-dioxin
AhR	aryl hydrocarbon receptor
HSCs	Hepatic stellate cells
ATRA	All-trans retinoic acid
MACS	magnetic-activated cell sorting
FACS	flow cytometric cell sorter
DCregs	regulatory DCs
Mregs	regulatory macrophages
NKT cells	Natural Killer T cells
iNKT cells	invariant NKT cells
